# Lateral Flow Microimmunoassay (LFµIA) for the Reliable Quantification of Allergen Traces in Food Consumables

**DOI:** 10.3390/bios12110980

**Published:** 2022-11-07

**Authors:** Amadeo Sena-Torralba, Javier Gabaldón-Atienza, Aitor Cubells-Gómez, Patricia Casino, Ángel Maquieira, Sergi Morais

**Affiliations:** 1 Instituto Interuniversitario de Investigación de Reconocimiento Molecular y Desarrollo Tecnológico (IDM), Universitat Politècnica de València, Universitat de València, Camino de Vera s/n, 46022 Valencia, Spain; 2 Departamento de Bioquímica y Biología Molecular, Universitat de València, Dr Moliner 50, 46100 Burjassot, Spain; 3 Instituto Universitario de Biotecnología i Biomedicina (BIOTECMED), Universitat de València, Dr Moliner 50, 46100 Burjassot, Spain; 4 Group 739 of the Centro de Investigación Biomédica en Red sobre Enfermedades Raras (CIBERER) del Instituto de Salud Carlos III, 28220 Madrid, Spain; 5 Departamento de Química, Universitat Politècnica de València, Camino de Vera s/n, 46022 Valencia, Spain

**Keywords:** lateral flow immunoassay, nanoparticles, multiplexing, microarray, internal calibration, smartphone, food-borne allergens

## Abstract

Quality assurance and food safety are of great concern within the food industry because of unknown quantities of allergens often present in food. Therefore, there is an ongoing need to develop rapid, sensitive, and easy to use methods that serve as an alternative to mass spectrometry and enzyme-linked immunosorbent assay (ELISA) for monitoring food safety. Lateral flow immunoassay is one of the most used point-of-need devices for clinical, environmental, and food safety applications. Compared to traditional methods, it appears to be a simple and fast alternative for detecting food allergens. However, its reliability is frequently questioned due to the lack of quantitative information. In this study, a lateral flow microimmunoassay (LFµIA) is presented that integrates up to 36 spots in microarray format in a single strip, providing semi-quantitative information about the level of allergens, positive and negative controls, internal calibration, and hook effect. The LFµIA has been evaluated for the on-site simultaneous and reliable quantification of almond and peanut allergens as a proof of concept, demonstrating high sensitivity (185 and 229 µg/kg, respectively), selectivity (77%), and accuracy (RSD 5–25%) when analyzing commercial allergen-suspicious food consumables.

## 1. Introduction

Food allergy is considered one of the most significant healthcare problems of the 21st century, with an estimated incidence of 250 million allergy sufferers worldwide and a 50% increase in prevalence across the past decade [1]. In terms of hospitalization, the statistics are even more overwhelming. Approximately 200,000 people require medical care for allergic reactions to food in the U.S. every year, resulting in 2000 hospitalizations and 150 deaths [2]. Food-borne anaphylaxis has increased by 380% in recent years; hence, it is unsurprising that the U.S. has experienced its most remarkable expansion ever, with a market volume of USD 2.4 billion in 2020 [3].

While there is no remedy to prevent food allergies, hypersensitive consumers still rely on avoiding the ingestion of allergenic substances. This fact has triggered the development of commercial assays for detecting food allergens [4,5]. However, these tests are commonly designed for individualized allergen determination, dramatically limiting their applicability in scenarios where the hypersensitive consumer is allergic to more than one type of food. As a case in point, almost one-third of allergy sufferers present multiple food allergies [6]. This circumstance represents a real bottleneck for the end user, who is advocated to buy different detection kits, apply several sample extraction protocols, and use diverse external calibrators, which cause high assay variability and low reliability.

In recent years, some of the developed biosensing approaches for food-borne allergen detection have been designed to analyze multiple allergens, ELISA and lateral flow immunoassay (LFIA) being the most relevant ones [7,8,9,10,11]. The former has a high sensitivity to various peanut allergens [12]. Still, the time-consuming assay procedure and the requirement for costly instrumentation hinder its application at the point-of-need. In contrast, the simplicity and cost-effectiveness of the latter empower its application in resource-limited settings [13]. LFIA has demonstrated a notable performance when undergoing multiplexing analysis with commercial products, as in the case of β-lactoglobulin and casein detection in cheese and milk samples. In this case, dyed latex particles are used as colorimetric reporters, generating red and blue signals in independent test lines [14].

**Figure 1:**
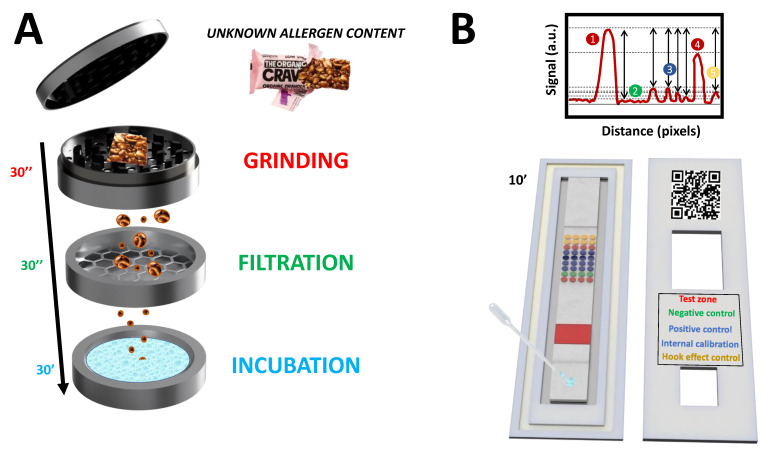
Schematic representation of (**A**) the portable device that contains all the elements required for the on-site extraction of the allergenic proteins. Specifically, these elements enable the grinding (30 s), filtration (30 s), and incubation of the sample with the extraction buffer for 30 min. (**B**) The proposed AuNPs-based LFµIA with microarray layout contains replicate assays for the in-situ detection of peanut and almond allergens, an internal signal calibration, and positive, negative, and hook effect control assays in 10 min.

Similarly, Anfossi et al. have proposed a multiplexed LFIA for detecting casein, ovalbumin, and hazelnut allergens in commercial biscuits, using AgNPs, spherical and desert-rose AuNPs. These nanoparticles generate a yellow, magenta, and cyan color code that facilitates the interpretation of the results by the non-experienced end user [15]. However, these strategies rely firstly on complex food sample extraction procedures and secondly on the performance of time-consuming external calibration curves for proper allergen quantification. Additionally, the limited information provided by these approaches prevents the identification of fundamental aspects of the assay, such as possible false negative/positive results due to the matrix and the hook effects. The matrix effect provokes an under- or overestimation of the amount of analyte in the sample. In contrast, the hook effect occurs when the amount of analyte in the sample exceeds the assay’s dynamic range. In this situation, detecting different analytes by the capture and detection bioreceptors hinders the generation of the immunorecognition event. Consequently, a decrease in signal response is obtained, resulting in an underestimation of the amount of analyte in the sample. To this end, the present work aims to develop an innovative LFIA for the multiplexed detection and quantification of peanut and almond allergens as a proof of concept because these allergens are responsible for almost 30% of food allergies [16]. The novelty of the proposed biosensing approach relies on the use of a power-free and fast allergen extraction method and an LFIA with a microarray layout that includes up to 36 spots within a single strip (Figure 1). Eight spots are devoted to detecting the allergenic proteins; 16 are positive controls and act as internal calibrators for fast and cost-effective assay quantification. Four spots are negative controls, and the remaining eight evaluate the hook effect. No academic or commercial biosensing approach provides such information in a single assay step with direct applicability in point-of-need scenarios. Thus, it is envisaged that the developed lateral flow microimmunoassay (LFµIA) will inspire the scientific community and the industry to create novel biosensing platforms with enhanced multiplexing capabilities and reliability.

## 2. Experimentalal Procedure

### 2.1. Materials, Reagents, and Instruments

Materials, reagents, and instruments are detailed in the supporting information.

### 2.2. Protocol of Allergen Extraction, Quantification, and Characterization

Food proteins were extracted from crude almonds and peanuts using the following procedure. First, 2 g of peanuts and almonds were shredded with a mixer and defatted by incubating the shreds in 10 mL of hexane for 1 h. Then, the hexane was removed using a rotary evaporator (360 mbar, 40 °C, 1 h), and the shreds were incubated with the extraction buffer (Tris 50 mM, pH 7.4) in a rocker (70 rpm) for 30 min at room temperature. Finally, the extract was centrifuged at 4200 rpm for 30 min, and the supernatant was filtered (0.45 µm) and stored at −20 °C until use. The total protein content of the extracts was quantified using the Bradford method [17]. The protein extracts were characterized by 15% SDS-PAGE gel electrophoresis (200 V for 40 min).

### 2.3. Polyclonal Antibodies Production, Purification, and Characterization

The polyclonal antibodies against the almond and peanut protein extracts were raised in New Zealand White rabbits [18]. The anti-sera titration was performed by ELISA, fixing 100 µL of the protein extracts (10 µg/mL in carbonate buffer (10 mM, pH 9.6)) and incubating 100 µL of serial dilutions of the anti-sera (1/2000-1/1,024,000) in PBST (10 mM, pH 7.4, 0.05% Tween-20)) and 100 µL of HRP-conjugated anti-rabbit IgG (0.2 µg/mL in PBST) for 1 h at room temperature. The sera were purified using a HiTrap^TM^ Protein G HP purification column, and the total IgG was measured using a Nanodrop 2000. A western blot analysis was performed using 0.5 µg/mL and 0.2 µg/mL of the primary and secondary antibodies, respectively, to identify the reactivity of the purified antibodies.

### 2.4. AuNPs’ Bioconjugation and Strips Fabrication

The AuNPs were synthesized following the Turkevich method [19] and characterized by electron transmission microscopy (TEM) and spectrophotometry. The AuNPs were functionalized with purified polyclonal antibodies and fixed on glass fiber, following the procedure reported by Parolo et al. [13]. The capture bioreceptors were dispensed in microarray format (4 × 9) on the nitrocellulose membrane using a Biodot dispenser (dispensing volume of 25 nL per spot in PBS 10 mM, pH 7.4). The antibodies against almond and peanut proteins were dispensed at 2.0 mg/mL, the BSA (negative control) at 1.0 mg/mL, the anti-rabbit IgG (internal control) at 0.75, 0.50, 0.25, and 0.12 mg/mL and the almond and peanut proteins (hook spots) at 2.0 mg/mL. The capture bioreceptors were fixed by incubating the membrane for two hours at 37 °C. Then, the membrane was blocked with BSA, as reported by [20]. The membranes were assembled onto a laminated card and cut into strips of 6 mm width using a strip cutter.

### 2.5. LFIA Procedure and Evaluation

The calibration curve for the multiplexed detection of almond and peanut allergens was performed by adding 100 µL of serial dilutions of the protein extracts (0, 10, 30, 100, 300, 1000, 3000, and 10,000 ng/mL in PBST) to the sample pad. The assay was evaluated qualitatively by visually inspecting the signal intensity in the TL 10 min after the sample addition. In addition, it was evaluated quantitatively by taking photographs using a smartphone (at a distance of 9 cm from the strip) and analyzing these with Image J [13]. The signal intensity in the spots related to the first, second test zone, and hook effect control zones was normalized following this equation: (ZITL—BG)/(PCCL—BG), where ZITL, PCCL, and BG were the signal intensities of the zone of interest, test line, positive control (spots 3A, 4B, 5C, 6D) control line, and background, respectively. The background corresponds to the area of the detection pad where there are no immobilized spots. We have selected a zone 1 mm below the first test zone. The positive control is used for normalization since the signal response is constant and independent of the concentration of the target analyte. A min–max normalization was performed to compare better the signals of the almond and peanut calibration curves and the positive and negative controls. The detection limit was calculated as the analyte concentration corresponding to the mean OD of the blank + 3SD (ten blank replicates). The limit of detection was calculated as the analyte concentration corresponding to the mean OD of the blank + 10SD (10 blank replicates) [21]. The proper dynamic range was measured considering the range in which the sensor response transits from 10% to 90% of its signal output [22].

### 2.6. Testing Commercial Food Samples

The assay was tested to evaluate the allergenic content of commercial snacks. The protein extraction was performed following the proposed method, using a portable grinder. Two grams of the snack were ground for 30 s. Then, 6 mL of the extraction buffer (Tris 50 mM, pH 7.4) was introduced into the grinder’s storage compartment, where the shreds were stored. The shavings were incubated with the extraction buffer for 30 min at room temperature. Finally, 110 μL of the sample extract was added dropwise to the strip’s sample pad. The proposed extraction method was compared with the commercial procedure reported by R-biopharm [23].

## 3. Results and Discussion

### 3.1. Characterization of the Protein Extracts and Antibodies

The total protein content was quantified using the Bradford method. The protein content was 8.6 and 5.9 mg/mL for the almond and peanut extracts, respectively. Then, SDS-PAGE electrophoresis was performed to identify the allergenic proteins and evaluate the effect of the extraction procedure on the protein’s stability. The protein profiles achieved for the almond and peanut extracts were similar to the reported ones. As observed in Figure S1, up to 25 different protein bands, ranging from 12 to 66 KDa, were identified in the almond extract, the 20–22 and 38–42 KDa ranges being the most abundant. These two proteins are known to form the amandin protein (the most abundant protein in almonds, 360 KDa) when bonded together [24,25]. Moreover, out of the 25 proteins present in almonds, eight of them have been reported as allergenic [26]. Eight allergens were identified in the almond extract (Table S1). Regarding the peanut extract, the three major allergenic proteins were also identified: Ara h 1, Ara h 2, and Ara h 3, which have a molecular weight of 65, 17, and 61 KDa, respectively [27]. The latter is based on two subunits corresponding to the bands at 45 and 22 KDa [28].

Once the presence and stability of the allergenic proteins in the food extracts were confirmed, polyclonal antibodies were raised by immunizing the rabbits with the respective extracts. The immunization process was monitored by evaluating the antibody titer with ELISA. A titer of 1/10^5^ and 1/6.1·10^7^ was achieved for the almond and peanut anti-sera, respectively, denoting a successful production of specific antibodies (Figure S2). Furthermore, a western blot was performed to identify the proteins in the food extract that present reactivity against the raised antibodies. As shown in Figure S3, the antibodies recognize the major allergenic and non-allergenic proteins. This fact is of interest in the food industry, considering the current heterogeneous and rapidly changing hypersensitivity dispersion.

### 3.2. Development of Individual Assays

Single non-competitive (immunosandwich) assays were developed to evaluate the sensitivity and selectivity of the assay using the purified antibodies. The LFIA followed the conventional double line (test and control lines). AuNPs of 20 nm sizes were selected as the signal transducer (Figure S4) due to their simple, cost-effective synthesis and straightforward bioconjugation via direct adsorption of the antibodies to the nanoparticle’s surface. Calibration curves were performed by interrogating serial dilutions of known concentrations of the extracted proteins. The collected data for each calibration curve was fitted to a four-parameter logistic (sigmoidal) equation (Figures S5 and S6), and the analytical parameters related to the LoD, LoQ, IC_50_, dynamic range, and linear regression coefficient (r^2^) were determined (Table S2). Both calibration curves followed a dose-response relationship within the ng range, which is more than enough, considering that the eliciting dose predicted to provoke a reaction in 50% of individuals (ED_50_) with an almond allergy- and peanut-derived proteins is 20 and 29 mg, respectively [29].

### 3.3. Design of the LFµIA Layout

**Figure 2:**
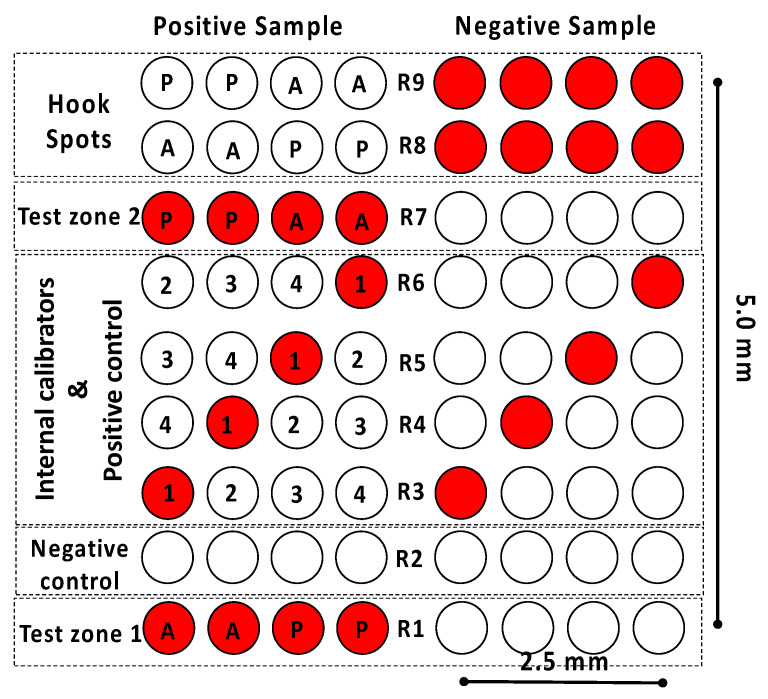
Schematic representation of the microarray layout, representing the expected results of positive and negative samples. In test zones 1 and 2, the antibody against almond (A) and peanut proteins (P) are immobilized in rows R1 and R7, respectively, bovine serum albumin (negative control) in row R2, the secondary antibody against rabbit IgG (internal calibrators 1–4) in rows R3–R6, and almond and peanut proteins in rows R8 and R9 (hook spots).

Once the developed LFIAs had an appropriate sensitivity when detecting the allergenic proteins of interest, the next step was to design the microarray layout. As previously discussed, the idea was to (1) integrate the almond, and peanut assays into the same strip and (2) provide the end user with more analytical information than the conventional LFIA. To this end, assay duplicates were included in the microarray to detect almond- and peanut-extracted proteins (row R1), together with four replicates of a negative control assay (row R2), four replicates of an internal calibration curve, and a positive control assay (rows R3–R6). Duplicates of a second test zone for almond and peanut (row R7) and four replicates of a hook control assay (rows R8–R9) were also included (Figure 2).

At this stage, the main objective was to miniaturize the microarray to fit 36 spots within a reduced sensing area. According to previous experience and the existing published advice regarding the design and development of LFIA [13], the first row was printed 15 mm from the conjugate pad, providing enough interaction time for the immunocomplex formation between the conjugated AuNPs and the target analyte. Next, the diameter of the spots was optimized by evaluating different dispensed volumes (25, 50, and 75 nL). As expected, the diameter of the spots enlarged as the volume increased. The average diameter of the spots was 0.30 ± 0.03 mm, 0.40 ± 0.05 mm, and 0.38 ± 0.08 mm when dispensing 25, 50, and 75 nL, respectively (Figure S7A).

Interestingly, it was also observed that the dispensing reproducibility significantly decreased when spotting 50 and 75 nL. The percentage relative standard deviation of the spots’ diameter was 8.9, 12.4, and 20.0% when dispensing 25, 50, and 75 nL, respectively. Analysis was also carried out on how the diameter of the spots affects the signal generated when performing LFIA. To this end, calibration curves were performed based on a direct immunoassay format by fixing the concentration of the secondary anti-rabbit IgG (0.12, 0.25, 0.50, 0.75, and 1.00 mg mL^−1^ in PBS) in the nitrocellulose membrane of the LFIA strips. AuNPs conjugated with the purified antibodies were used as the biosensing element. As shown in Figure S7B, the highest signal, analytical sensitivity, and regression coefficient were achieved when dispensing 25 nL of the capture bioreceptors. This result might be due to the high density of bioreceptors in the spots, which increases the number of AuNPs per mm^2^. Moreover, the spots produced when dispensing 25 nL can be visualized by direct naked-eye inspection, since there is a proper separation distance of 0.28 ± 0.02 mm between other spots. Consequently, supported by the above results, 25 nL was selected as the optimal dispensing volume.

The microarray had dimensions of 5 × 2.5 mm and covered one-third of the detection pad in length and two-thirds in width. The arrangement of the various elements in the microarray was carefully designed. The test zone (spots) must be placed in row R1 to ensure that the differences in the generated signal are related to the concentration of the allergenic proteins rather than the differences in the number of available conjugated AuNPs. Thus, based on the selected non-competitive assay format, a positive sample containing proteins of both almond and peanut will trigger the generation of a high signal in the test zone. In contrast, a low signal will be related to a negative sample.

Similarly, the negative control assay must be placed as close as possible to the test zone, which in this case was in row R2. The reason is that the excess of conjugated AuNPs in this zone is still high. Hence, achieving a negligible signal in the negative control assay (fixed bovine serum albumin) proves that the conjugated AuNPs do not generate non-specific signals.

As far as the internal calibration assay is concerned, the signals come from the immobilized secondary anti-rabbit IgG (0.75, 0.50, 0.25, and 0.12 mg mL^−1^ as calibrators 1 to 4, respectively). Consequently, the signal generated will be high, regardless of the concentration of target analytes in the sample; this is why the internal calibration is also used as a positive control assay. The signal intensities will vary linearly, depending on the concentration of the secondary antibody immobilized on the nitrocellulose membrane. The analyte concentration in the sample will be quantified by comparing the signal intensity of the internal calibrators with the one in the test zone. Four replicates of each point were used to increase the internal signal calibration accuracy. The spots corresponding to the four concentrations were included in each row and exchanged in each replicate. The purpose of the latter is to check that there are no differences in the signal intensity when placing a spot on the left or the right of the microarray since this would indicate that the sample flow is not homogeneously distributed along the transverse axis of the LFIA strip.

Test zone 2 (row R7) is a replicate of test zone 1 (row R1) and aims to detect the almond and peanut proteins that cannot react at row 1, mainly due to the lack of interaction time or the saturation of the capture antibodies. Considering the lower amount of available AuNPs at this point of the LFIA strip, the second test zone could provide a shift in the dynamic range compared to the one achieved in the first test zone. In this way, more qualitative information for the end user is obtained. The spots related to the almond and peanut assay were also exchanged to check if the sample was flowing homogeneously. Finally, the hook effect control zone corresponds to rows 8–9. The hook effect is a false negative result produced by an extremely high concentration of the target analyte in the sample [30]. Four replicates of almond and peanut proteins were included in the array layout to study this hook effect. Increasing target analyte concentrations in the sample will reduce the available detection antibodies conjugated to the AuNPs, which translates to progressively lower signal intensities in the hook effect control zone. In this sense, the lack of signal in the test zones will be attributed to the hook effect and not to the interrogation of a negative sample, provided that there is no signal in the hook effect control zone.

### 3.4. Development of Multiplexed Assays in LFµIA

**Figure 3:**
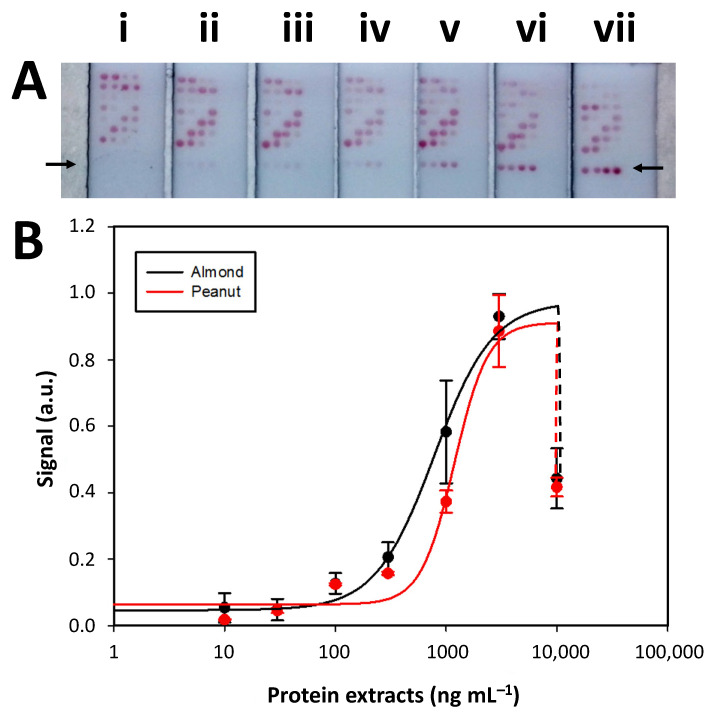
(**A**) Picture of the LFµIA strips after simultaneously detecting (i) 0, (ii) 10, (iii) 30, (iv) 100, (v) 300, (vi) 1000, and (vii) 3000 ng mL^−1^ of almond- and peanut-extracted proteins. (**B**) Normalized (min–max) calibration curves obtained when quantifying the mean signal generated in test zone 1 (row R1). Error bars show the standard deviation of 3 replicates.

The next step involved calibration curves to simultaneously detect almond- and peanut-extracted proteins. The signal intensity in the test zones increased with higher target analyte concentrations. Signal saturation was reached at 3000 ng mL^−1^ before experiencing a decrease due to the hook effect. The signal intensities generated in the first test zone (row 1, indicated with an arrow in Figure 3A) were normalized (min–max), fitted to a four-parameter logistic (sigmoidal) equation (Figure 3B), and the analytical parameters related to the LoD, LoQ, IC_50_, dynamic range and linear regression coefficient (r^2^) were determined (Table S3). The achieved sensitivity expressed as LoD was 185 and 229 µg/kg for the almond and peanut assay, respectively. Compared with the analytical parameters achieved with the individual calibration curves in the conventional LFIA format (Section 3.2), the almond assay showed slightly lower sensitivity and a shift of the IC_50_ and dynamic range to higher concentrations of a target analyte. Conversely, the peanut assay presented a similar LoD, LoQ, IC_50,_ and dynamic range. The calibration curve’s relative standard deviations (RSD) were between 5% and 25% within 100 to 3000 µg/mL for both analytes. Thus, it was concluded that integrating the almond and peanut assays within the same strip in a microarray format did not significantly vary the analytical performances.

The signal intensities related to the second test zone were then analyzed. As expected, these were much lower than their counterparts in the first test zone due to the fewer available conjugated AuNPs and target analytes. However, the study took advantage of this condition to develop a test zone with a right-shifted dynamic range. Following the ratiometric concept [31], when combining the same concentration of immobilized capture bioreceptors with lower amounts of conjugated AuNPs, a much higher target analyte concentration will be required to achieve a dose-response relationship. This approach enabled an 86.8 and 47.2% higher IC_50_ in the almond and peanut assays, respectively, of the second test zone compared with the first test zone (Table S3). This shift in the dynamic range was beneficial since it overcame the hook effect observed when evaluating 3000 ng mL^−1^ of the target analyte in the first test zone (Figure S8). Moreover, the dynamic range of the second test zone for the almond and peanut assays also covered a 3.4- and 1.5-fold higher concentration of target analyte compared to the first test zone. To this end, using the first and second test zones provides an enhanced amount of information because they cover a wider dynamic range than a single test zone. Moreover, the degree of shift in the dynamic range of the second test zone can be modulated by the immobilization of higher or lower concentrations of capture antibodies.

### 3.5. Assay Selectivity

**Figure 4:**
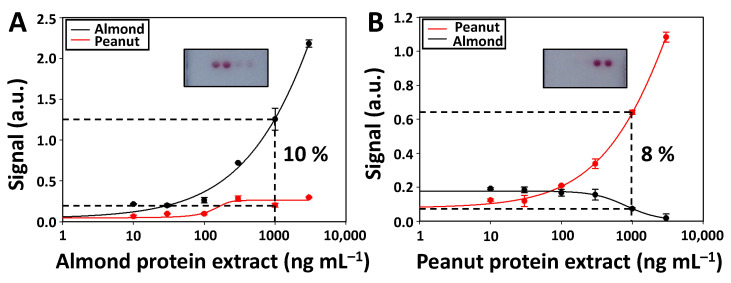
Cross-reactivity (%) between the assays corresponding to the first test zone (row 1) when analyzing 1000 ng mL^−1^ of (**A**) almond and (**B**) peanut protein extract. Inset showing the picture of the first test zone (row 1) after performing the cross-reactivity assay with 1000 ng mL^−1^ of almond and peanut protein extract. The signals correspond to the mean value of 3 replicates, while the error bars show the standard deviation.

One of the most important aspects when developing multiplexed biosensing platforms is to assess the assay selectivity, as this parameter indicates the degree of cross-reactivity between the antibodies and target analytes of the different assays. A high selectivity ensures that the signal achieved in the test zone is related to detecting the desired target analyte. Therefore, calibration curves were performed for the individual detection of almond (Figure 4A) and peanut (Figure 4B) proteins using LFµIA, and the signal intensities were measured in the spots of the first test zone (Figure 4 inset). As observed, a dose-response relationship was only achieved in the assays of each particular analyte. In contrast, the other assays show a low and constant signal independent of the concentration of proteins. The percentage cross-reactivity between the assays was quantified by dividing the signal intensity achieved when detecting the proteins of interest with the non-intended match pair by the one achieved with the intended match pair [32]. The cross-reactivity of the assay was calculated by using 1000 ng mL^−1^ of each protein extract since this value is close to the IC_50_ in both assays. The peanut assay has a 10% cross-reactivity with the almond proteins, while the almond assay shows an 8% cross-reactivity with the peanut proteins. The obtained results determine that the developed multiplexed LFµIA is highly selective. The antibodies and target analytes related to almond and peanut assays practically do not cross-react.

The cross-reactivity of the developed LFµIA with other common allergenic proteins was also assessed, comparing the obtained signal response when analyzing other allergens with the expected response when analyzing the intended allergens. The cross-reactivity of the almond and peanut assays was lower than 23% when evaluating 1000 ng mL^−1^ of shrimp, hazelnut, egg, milk, wheat, and fish allergens (Figure S9 and Table S4). Furthermore, the signal recovery ($\frac{signal\;response\;prick\;test}{signal\;response\;protein\;extract}\times \;100$) when analyzing 1000 ng mL^−1^ of the almond and peanut prick test allergens was 110.3 ± 30.5% and 142.6 ± 13.8%, respectively. Such high percentage signal recoveries reveal strong analytical performances of LFµIA.

### 3.6. Assay Controls and Internal Calibration

**Figure 5:**
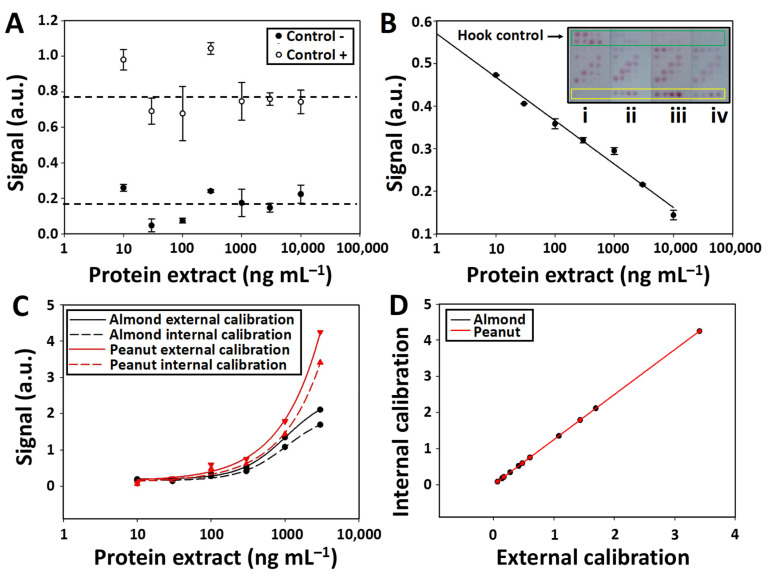
(**A**) Normalized (min–max) mean signal intensities in the positive (spots 3A, 4B, 5C, 6D) and negative (row R2) control assays. (**B**) Normalized mean signal intensities in the hook effect control assays (rows R8 and R9) when detecting serial dilutions of the protein extracts (10, 30, 100, 300, 1000, 3000, and 10,000 ng mL^−1^). (B inset) Picture of the strips after the detection (i) 0, (ii) 1000, (iii) 3000, and (iv) 10,000 ng mL^−1^ of the protein extracts. (**C**) Comparison of the mean signal ratio of the internal calibration curves (rows R3–R6) with the signal intensities of the external calibration curves (row R1) achieved when detecting serial dilutions of the protein extracts (10, 30, 100, 300, 1000, 3000, and 10,000 ng mL^−1^). (**D**) Mean signal intensity relationship between the internal and external calibration curves. Error bars show the standard deviation of 3 replicates.

After characterizing the analytical properties of the test zones, the next step was to analyze the information provided by the other elements of the microarray. Thus, the signal intensities in the negative and positive control assays were examined. This analysis confirmed that the values stayed constant (RSD < 4%), regardless of the extracted protein concentration added to the LFµIA strip. The high signal intensities in the positive control assay can be used to confirm that (1) the AuNPs are successfully conjugated to the purified antibodies and (2) the AuNPs conjugated solution flows appropriately along the nitrocellulose membrane (Figure 5A). On the other hand, the intensity of the negative control was negligible and similar to the background signal observed in the nitrocellulose membrane, thus proving the absence of non-specific signals.

The subsequent step involved analyzing the signal intensities generated in the hook effect control and fitting the data into a linear regression curve. As observed in Figure 5B, the signal intensity is inversely proportional to the concentration of the target analyte (O.I. = −0.044 ln [protein extract (ng mL^−1^)] + 0.57 R^2^ = 0.98). The hook effect control assays are direct immunoassays between the almond- and peanut-extracted proteins and the conjugated AuNPs. Thus, higher target analyte concentrations in the sample block a higher number of binding sites of the antibodies conjugated to the AuNPs, which hinders the biorecognition of the conjugated AuNPs by the immobilized proteins of the hook effect control zone. For that reason, the hook effect control assay is useful in situations like that observed in Figure 3B, where similar signal intensities are achieved when evaluating 1000 and 10,000 ng mL^−1^ (Figure 5B strips ii and iv). The differences in signal intensity (0.3 and 0.15, respectively) observed in the hook effect control assays of these two strips can be successfully used to distinguish both concentrations, thus reducing the occurrence of false results.

The signal intensities of the internal calibration spots were also measured alongside a comparison with those from the first test zone (external calibration) (Figure 5C). As shown in Figure 5D, the internal curve is similar to the external calibration (Table S5), revealing an accurate assay quantification in a single step.

### 3.7. Real Sample Analysis

The LFµIA was evaluated by testing a snack bar with a declared content of peanuts (34%) and possible traces of almonds. This testing allowed for (1) determining the percentage recovery when detecting peanuts and (2) checking whether the proposed biosensing platform had enough sensitivity to quantify traces of almonds. The protein extraction was performed using a fast, power-free method that can be easily performed on-site. A grinding device with dimensions of 4.8 ×4.5 cm was used, containing all the required elements for extracting the allergenic proteins from the sample. As shown in Figure S10, the method consisted of the manual grinding of the snack bar, followed by the filtering of the shreds, the addition and incubation of the extraction buffer, and the collection of the solution containing the extracted proteins. Compared with a standard commercial extraction approach, which requires bulky external instrumentation (water bath, rotatory station, and centrifuge) and at least seven different steps [23], the proposed method is 3.6-fold faster. It enables the extraction of 76.7% of the proteins (according to the Bradford method).

The next step was to analyze the extracted proteins with the developed LFµIA. As is shown in Figure S11i, low signal intensity in the almond assay was detected, alongside a significantly high signal intensity in the peanut assay. By comparing the achieved signals with the ones of the internal signal calibration curve, a concentration of almond proteins of 212 ± 45 ng mL^−1^ (RSD 21%) was determined, a value that was slightly lower than the LoQ (273.9 ng mL^−1^) but higher than the LoD (184.8 ng mL^−1^). Thus, the LFµIA could not provide a reliable value of the almond content, but the assay showed enough analytical sensitivity to detect traces of almonds. The detected quantity of peanut protein in the sample was 516 ng mL^−1^. However, the hook effect control assay showed a negligible signal intensity in the peanut assay, indicative of the hook effect due to the presence of peanut proteins at a concentration higher than 3000 ng mL^−1^. The analysis of the peanut protein content with the Bradford method confirmed a concentration of peanut proteins of 23,000 ng mL^−1^ (considering that the snack bar declared a 34% peanut content and that 26% of the peanut content is related to proteins). Therefore, it was proved that the developed LFµIA provided helpful information for identifying the hook effect. It is worth mentioning that, without the hook effect control assay, the performed assay would have returned a false negative result by quantifying a 45-fold lower concentration of peanut in the sample.

Then, the extracted sample was diluted to adjust the peanut protein content to 2300 ng mL^−1^, which is within the operating range of the calibration curve. This dilution enabled the proper quantification of the peanut protein content with the internal signal calibration of the LFµIA (Figure S11ii). The LFµIA results revealed a concentration of peanut protein of 1343 ng mL^−1^, corresponding to a recovery of 58 ± 8%. Considering that the Bradford method calculated the peanut protein concentration, this result can be regarded as appropriate. Finally, as expected, a decrease was observed in the signal intensity of the peanut assay, alongside a signal intensity increase in the hook effect control assay, when diluting the sample up to a peanut protein content of 230 ng mL^−1^ (Figure S11iii). These data revealed that this sample had a lower peanut protein concentration than the previously analyzed one. In quantitative terms, the peanut proteins were detected at a concentration of 451 ng mL^−1^, resulting in a 196% recovery. However, it is worth mentioning that the peanut protein content taken as reference (230 ng mL^−1^) was out of the dynamic range of the calibration curve (300–3000 ng mL^−1^) and lower than the LoQ (264.7 ng mL^−1^). The data revealed that the developed LFµIA could provide accurate qualitative information on the almond and peanut protein content and semiquantitative information when the protein concentration was within the dynamic range. Moreover, it is fundamental to highlight the appropriateness of the positive and negative control assays integrated into the LFµIA, since the signals remain constant when analyzing such a complex matrix.

## 4. Conclusions

A multiplexed microarray-based LFµIA has been developed to quantify almond and peanut allergenic proteins simultaneously. To this end, almond and peanut protein extracts were used to produce polyclonal antibodies, which showed good selectivity. The developed antibodies were used as capture bioreceptors in the LFµIA, displaying appropriate analytical properties and sufficient sensitivity to detect concentrations of almond and peanut allergens lower than the ED_50_ value.

The main novelty of this work is incorporating several elements in the microarray that aim to provide essential assay information, which simplifies the interpretation of the results by non-specialized end users. For instance, this work has proved that by including several replicates of negative and positive control assays, the end user can quickly identify false negative or positive assay results. Similarly, the hook effect control has proved useful when determining almond and peanut protein concentrations semi-quantitatively. Furthermore, internal signal calibration has been a highly accurate, faster, and less expensive approach for assay quantification than conventional external calibration. Finally, incorporating two test zones in different microarray sections has enabled the acquisition of calibration curves with distinct dynamic ranges, which has proved to overcome the hook effect. Considering a cost of 1€ per strip, the proposed biosensing platform has direct commercial relevance for allergen quantification in food samples. It is foreseen that the developed platform may have great potential, both in the food safety area for fast on-site allergen identification in commercial products and in the pharmacy to analyze the purity of the allergy prick tests. From a future perspective, the multiplexing capabilities of the LFµIA can also be increased by implementing assays for other allergens in the microarray, expanding the applicability of the developed biosensing approach. Additionally, a smartphone-based reader with integrated artificial intelligence software would be an excellent option to simplify the interpretation of the assay results by non-specialized end users [33,34].

## Supplementary Materials

The following supporting information can be downloaded at: https://www.mdpi.com/article/10.3390/bios12110980/s1, Figure S1: SDS-PAGE gel electrophoresis of the almond and peanut protein extracts. Figure S2: Anti-almond and anti-peanut serum ELISA titrations. Figure S3: Western blot analysis of the almond and peanut protein extracts. Figure S4: Transmission electron microscopy image of the synthesized AuNPs, showing a spherical shape and a diameter of 22 ± 3 nm. Figure S5: Calibration curve for the detection of almond extract. Inset of the linear regression. Figure S6: Calibration curve for the detection of peanut extract. Figure S7: Optimization of the spots immobilized in the detection zone. Figure S8: Normalized calibration curves when quantifying the signal generated in the first test zone (row R1) and second test zone (row R7) when analyzing serial dilutions of almond and peanut with the LFµIA. Figure S9: Normalized signal intensities achieved in the first test zone (row 1) of the LFµIA when analyzing 1000 ng mL^−1^ of shrimp, hazelnut, egg, milk, wheat, fish, almond, and peanut allergens. Figure S10: (A) Pictures of the proposed grinder-based extraction method. (B) The snack bar is grinded, (C) the shavings are filtered and incubated with the extraction buffer. Figure S11: Pictures of the LFµIA strips after the detection of (i) 23000, (ii) 2300 and (iii) 230 ng mL^−1^ of the protein solution extracted from a snack bar. Table S1: Almond and Peanut allergens. Table S2: Limit of detection, quantification, IC50, dynamic range (DR), and linear regression coefficient (r^2^) of the individual assay. Table S3: Limit of detection, quantification, IC50, dynamic range (DR), and linear regression coefficient (r^2^) of the multiplexed assay. Table S4: Cross-reactivity (%) of the almond and peanut assays when analyzing 1000 ng mL^−1^ of the prick test allergens. Table S5: Limit of detection (LoD), Limit of quantification (LoQ), IC50, dynamic range (DR) and linear regression coefficient (r2) of the internal calibration curve.
