# Supplementary material for: Lateral Flow Microimmunoassay (LFµIA) for the Reliable Quantification of Allergen Traces in Food Consumables
Source: Biosensors (Basel). 2022 Nov 7;12(11):980. doi: 10.3390/bios12110980 (PMC9688043; doi:10.3390/bios12110980)
Supplement: Supplementary file 1 [file biosensors-12-00980-s001.zip › biosensors-1966035-supplementary.pdf]

biosensors

MDPI

Lateral Flow Microimmunoassay (LFμIA) for the Reliable Quantification of Allergen Traces in Food Consumables

Amadeo Sena-Torralba [1,*], Javier Gabaldón-Atienza [1], Aitor Cubells-Gómez [1], Patricia Casino [2,3,4], Ángel Maquieira [1,5] and Sergi Morais [1,5,*]

1 Instituto Interuniversitario de Investigación de Reconocimiento Molecular y Desarrollo Tecnológico (IDM), Universitat Politècnica de València, Universitat de València, Camino de Vera s/n, 46022 Valencia, Spain
2 Departamento de Bioquímica y Biología Molecular, Universitat de València, Dr Moliner 50, 46100 Burjassot, Spain
3 Instituto Universitario de Biotecnología i Biomedicina (BIOTECMED), Universitat de València, Dr Moliner 50, 46100 Burjassot, Spain
4 Group 739 of the Centro de Investigación Biomédica en Red sobre Enfermedades Raras (CIBERER) del Instituto de Salud Carlos III, 28220 Madrid, Spain
5 Departamento de Química, Universitat Politècnica de València, Camino de Vera s/n, 46022 Valencia, Spain
* Correspondence: asentor@upvnet.upv.es (A.S.-T.); smorais@upv.es (S.M.)

Keywords: Lateral flow immunoassay; Nanoparticles; Multiplexing; Microarray; Internal calibration; Smartphone; Food-borne allergens.

Reagents. Polyclonal goat to rabbit IgG (R5506), Freund's adjuvant complete (F5881) and incomplete (F5506), Bovine serum albumin (BSA) (A9418), Sucrose (S0389), Sodium tetraborate (S9640), Sodium citrate (71497), Tetrachloroauric (III) acid (520918), Trizma-HCl (T3253), Trizma-BASE (T1503), Hydrochloric acid, 37% (320331), SIGMAFAST™ OPD (P9187), Hydrogen peroxide solution (H1009), Skim Milk Powder (70166) and Phosphate buffered saline tablets (P4417) were purchased from Sigma Aldrich. Goat anti-Rabbit IgG H&L (HRP) preadsorbed (ab97080) was purchased from Abcam. Tween-20 (TW00201000) and Ethanol 96% v/v (ET0003005) were purchased from Scharlab. Boric acid (131015) was purchased from Panreac. Bradford protein assay dye reagent concentrate Cat. No. 5000006), TGX acrylamide solutions FastCast, Prestained protein ladder (1610373) and Clarity™ Western ECL Substrate (1705060) were purchased from Biorad. Hexane (HE0220), Sodium carbonate anhydrous (SO0116) and Sodium hydrogen carbonate (SO0131) were purchased from Scharlau. Crude almonds, peanuts, and nuts bar (barritas de frutos secos) were purchased from Mercadona. Skin prick tests were purchased from Leti pharma.

Materials. Glass fiber (GFCP000800), Cellulose fiber (CFSP173000) and Nitrocellulose membrane (Hi-FlowTM Plus 135) were purchased from EDM Millipore. Antibody purification column (HiTrapTM Protein G HP) was purchased from Cytiva lifesciencies. Protein purification filters (Amicon Ultra 0,5 ML 100 K) were purchased from Merck. ELISA plates (96-well Clear Flat Bottom Polystyrene Treated and Non-treated Microplate) were purchased from Corning. Nitrocellulose Membrane, 0.45 μm was purchased from BioRad.

Instruments. pH meter (Seven CompactTM) and analytical balance (XP205) were purchased from Metler Toledo. Bioreagent dispenser (AD1500) and strips cutter (CM4000) were purchased from BioDot. Rocker (Gyro rocker SSL3) was purchased from Stuart. Hot plate (Agimatic-N) and centrifuge (Medifriger-BL-S) were purchased from J.P. Selecta. Vacuum chamber was purchased from Kartell. Spectrophotometer (nanodrop 2000) was purchased from ThermoFisher Scientific. Plate reader (Victor 1420) were purchased from PerkinElmer. Oven was purchased from Memmert. Rotary evaporator (R-100) was purchased from Buchi. Mini-PROTEAN Tetra Handcast Systems and PowerPac Universal Power Supply were purchased from BioRad. Portable grinder was purchased from iLC. Microscope electron microscopy (FEI Tecnai G2 20) has been used at the UPV microscopy division. The LFA quantitative evaluation has been performed using a Huawei P30 lite smartphone and Image J software.

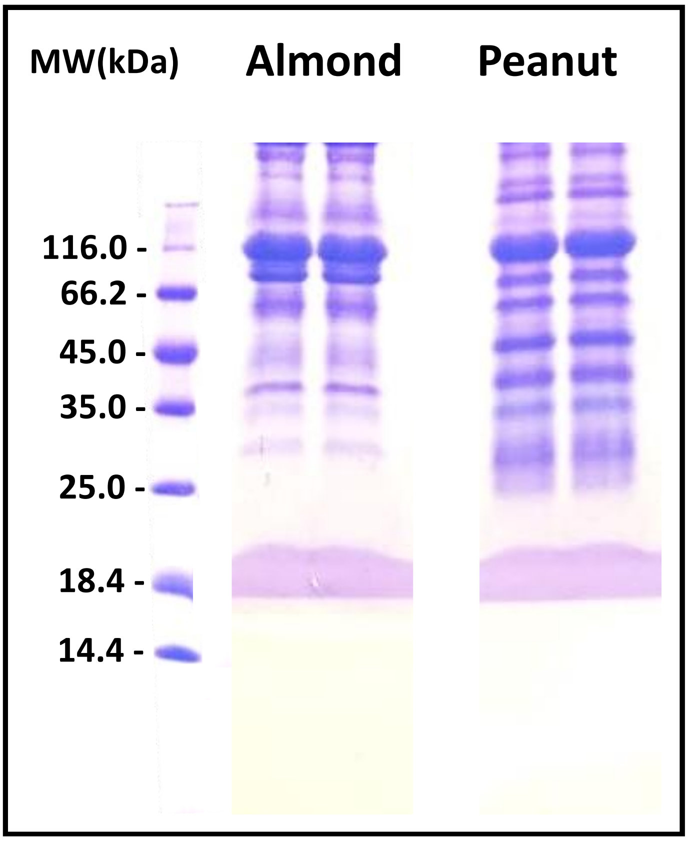

Figure S1. SDS-PAGE gel **electrophoresis** of the **(1-2)** almond and **(3-4)** peanut protein extracts.

Table S1. Almond and Peanut allergens

Almond allergens	Molecular weight (KDa)	Peanut allergens	Molecular weight (KDa)
Pru du 1	17	*Ara h 1*	65
Pru du 2	23-27		
Pru du 2S	12	*Ara h 2*	17
Pru du 3	9		
Pru du 4	14	*Ara h 3*	61
Pru du 5	10		
Pru du 6 (Amandin)	360	*Arachin (acidic)*	45
Pru du γ-conglutin	45	*Arachin (básic)*	22

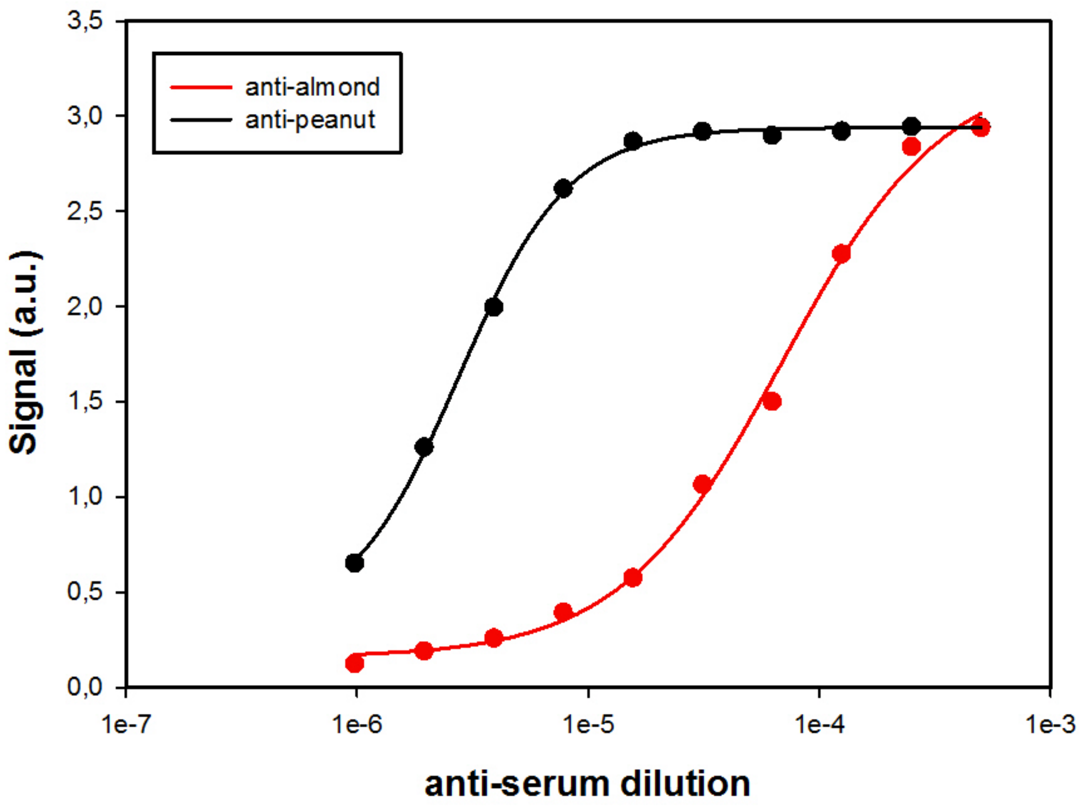

Figure S2. Anti-almond (red) and anti-peanut (black) serum ELISA titrations.

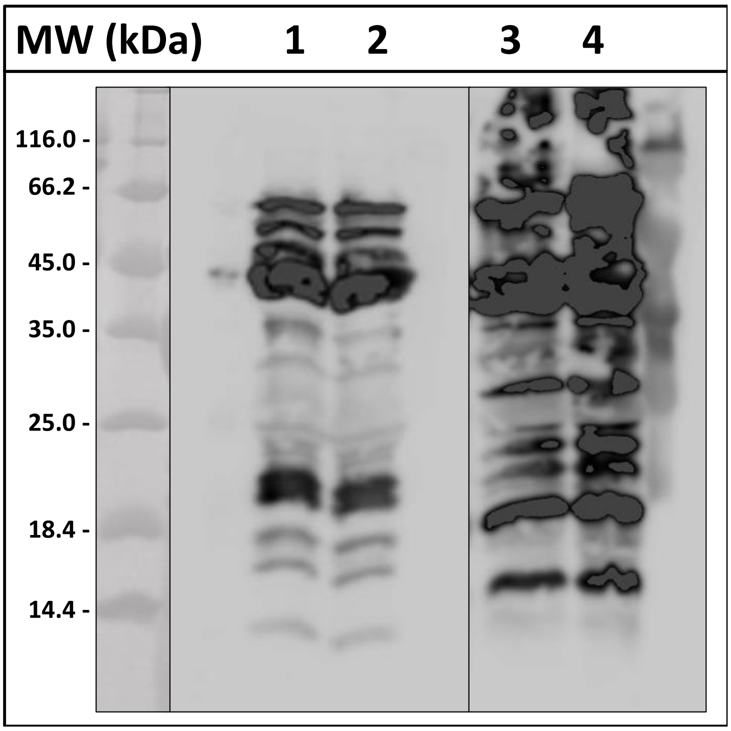

Figure S3. Western blot analysis of the **(1-2)** almond and **(3-4)** peanut protein extracts.

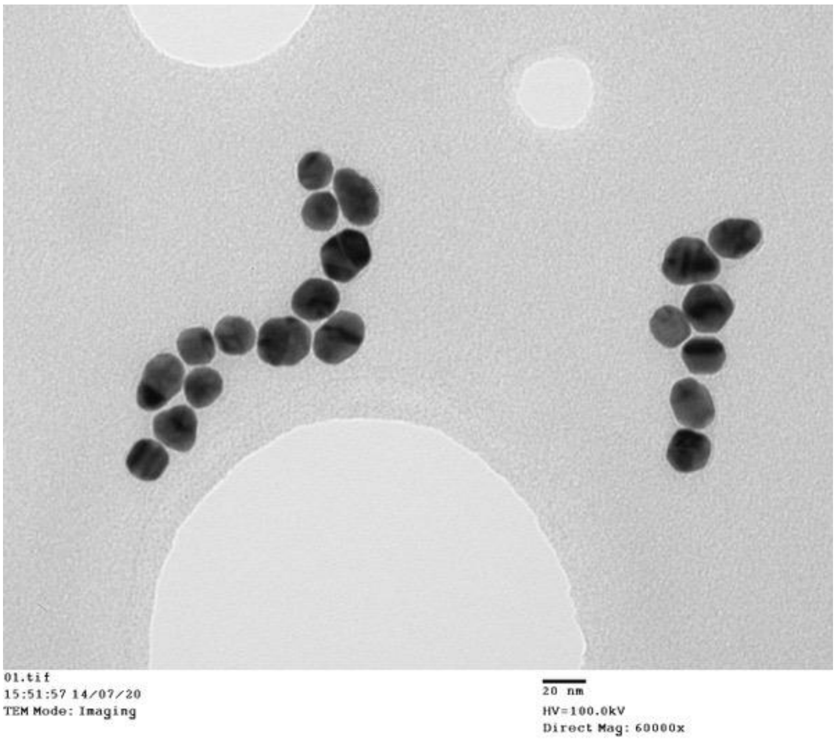

Figure S4. Transmission electron microscopy (TEM) image of the synthesized AuNPs, showing a spherical shape and a diameter of 22 ± 3 nm.

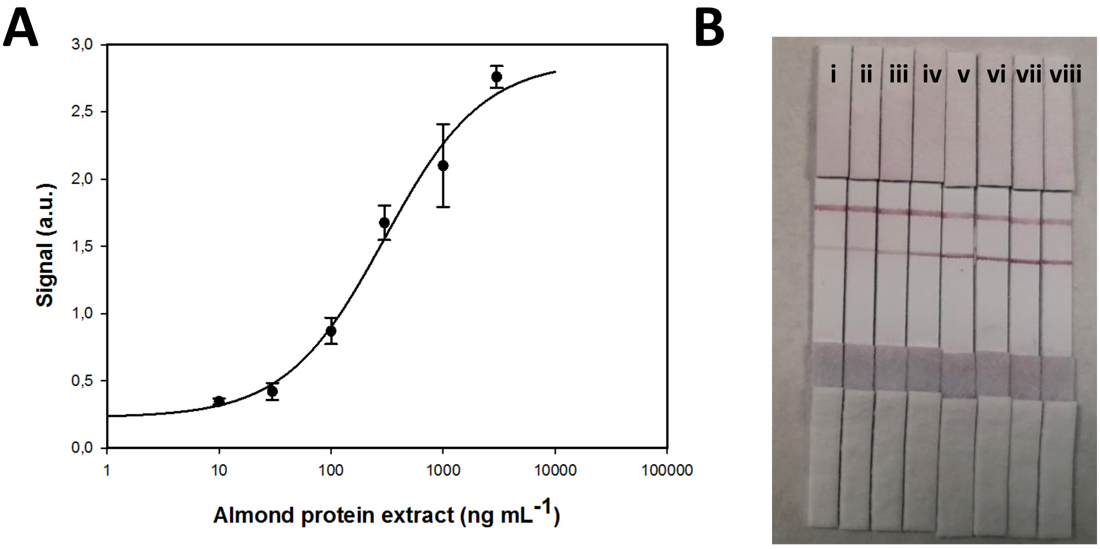

Figure S5. (A) Calibration curve for the detection of almond extract. Inset of the linear regression (O.I. = 0.512 ln [almond extract (ng mL^{-1})] – 1.369 R^2=0.98). **(B)** Picture of the LFIA strips after performing the assay for the detection of **(i)** 0, **(ii)** 10, **(iii)** 30, **(iv)** 100, **(v)** 300, **(vi)** 1000, **(vii)** 3000 y **(viii)** 10.000 ng/mL.

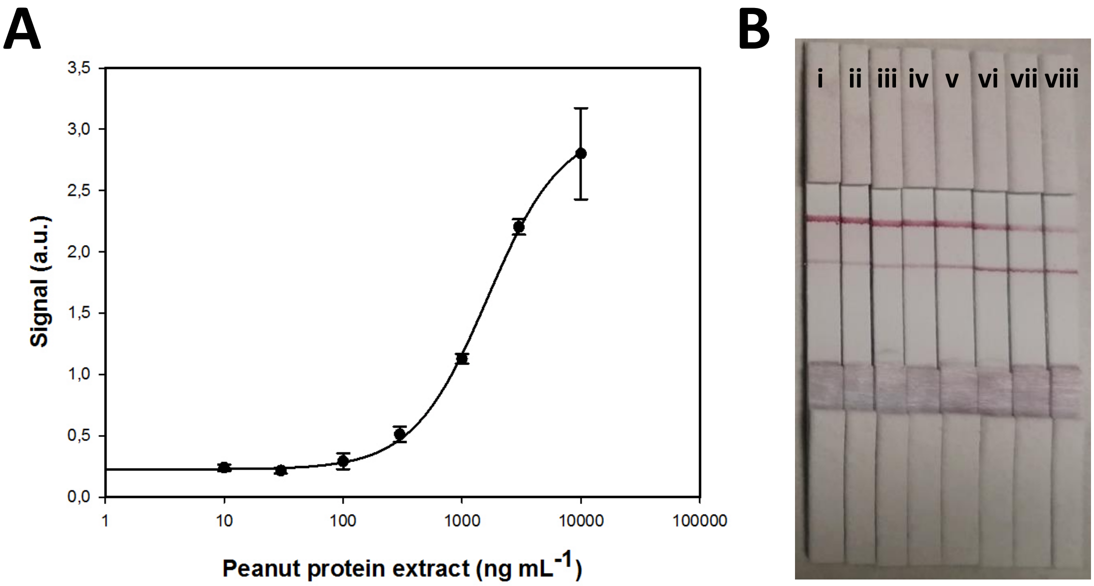

Figure S6. (A) Calibration curve for the detection of peanut extract. Inset of the linear regression (O.I. = 0.628 ln [peanut extract (ng mL^{-1})] – 3.425 R^2=0.98). **(B)** Picture of the LFIA strips after performing the assay for the detection of **(i)** 0, **(ii)** 10, **(iii)** 30, **(iv)** 100, **(v)** 300, **(vi)** 1000, **(vii)** 3000 y **(viii)** 10.000 ng/mL.

Table S2. Limit of detection (LoD), Limit of quantification (LoQ), IC50, dynamic range (DR) and linear regression coefficient (r^2) of the individual assay.

	LoD (ng mL^{-1})	LoQ (ng mL^{-1})	IC_{50} (ng mL^{-1})	DR (ng mL^{-1})	r^2
**Almond**	42.5	174.8	213.9 ± 16.9	25-1842	0.98
**Peanut**	222.5	272.9	1205.2 ± 304.2	219-6110	0.98

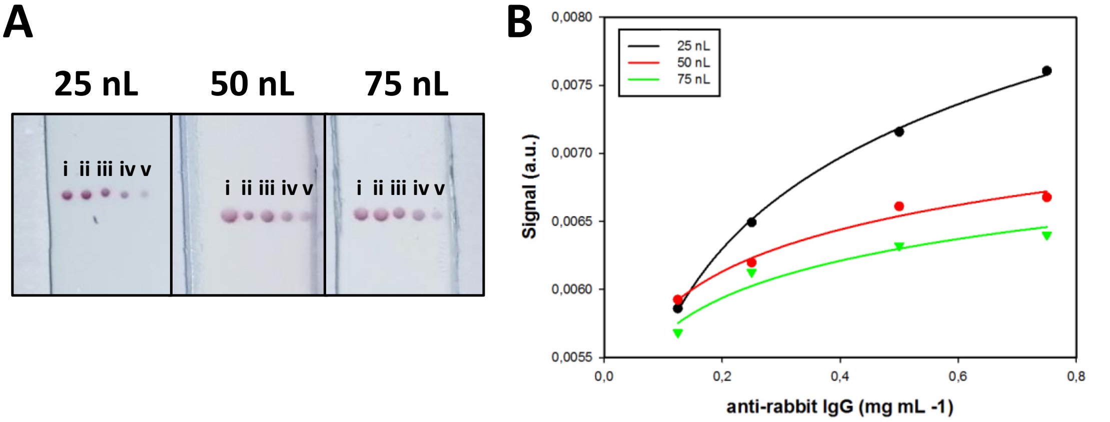

Figure S7. Optimization of the spots immobilized in the detection zone**. (A)** Picture of the strips after the detection of **(i)** 0.125, **(ii)** 0.250, **(iii)** 0.500, **(iv)** 0.750 and **(v)** 1.000 mg/mL of anti-rabbit IgG by direct assay when dispensing them in 25, 50 and 75 nL. **(B)** Calibration curve when dispensing the anti-rabbit IgG in 25 nL (O.I. = 0.001 ln [anti-rabbit IgG (mg mL^{-1})] + 0.0079 R^2=0.99), 50 nL (O.I. = 0.0004 ln [anti-rabbit IgG (mg mL^{-1})] + 0.0068 R^2=0.98) and 75 nL (O.I. = 0.0004 ln [anti-rabbit IgG (mg mL^{-1})] + 0.0066 R^2=0.94).

Table S3. Limit of detection (LoD), Limit of quantification (LoQ), IC50, dynamic range (DR), and linear regression coefficient (r^2) of the multiplexed assay.

Zone	Assay	LoD (ng mL^{-1})	LoQ (ng mL^{-1})	IC_{50} (ng mL^{-1})	DR (ng mL^{-1})	r^2
1	**Almond**	184.8	273.9	687.7 ± 74.6	100-2645	0.99

	**Peanut**	228.9	264.7	1175.1 ± 180.2	132-2799	0.98
**2**	**Almond**	761.3	2029.6	5212.3 ± 533.8	1293-10000	0.98
	**Peanut**	604.9	1289.2	2228.9 ± 208.8	362-4362	0.95

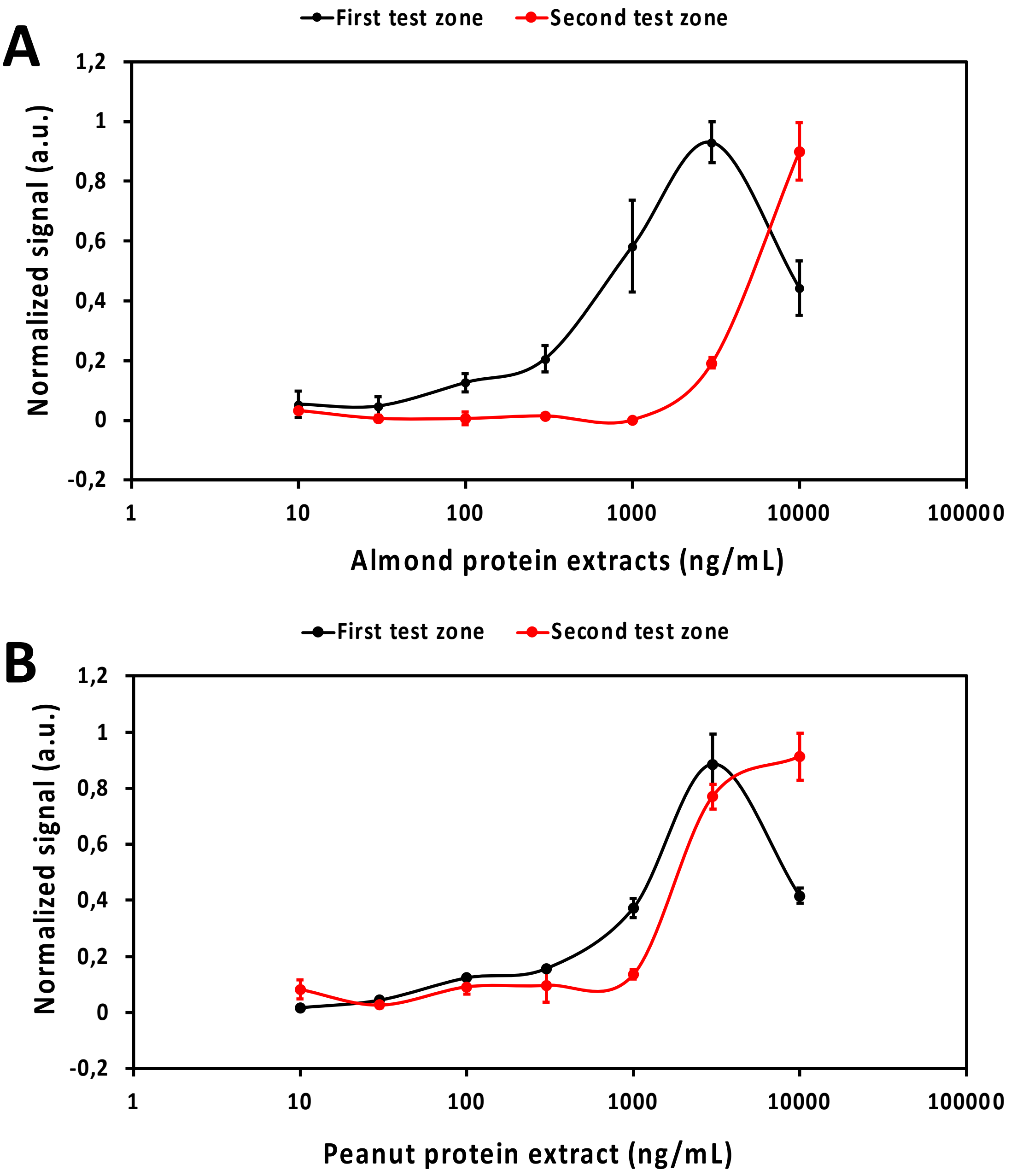

Figure S8. Normalized calibration curves when quantifying the signal generated in the first test zone (row 1) (black) and second test zone (row 7) (red) when analyzing serial dilutions of **(A)** almond and **(B)** peanut with the LFμIA.

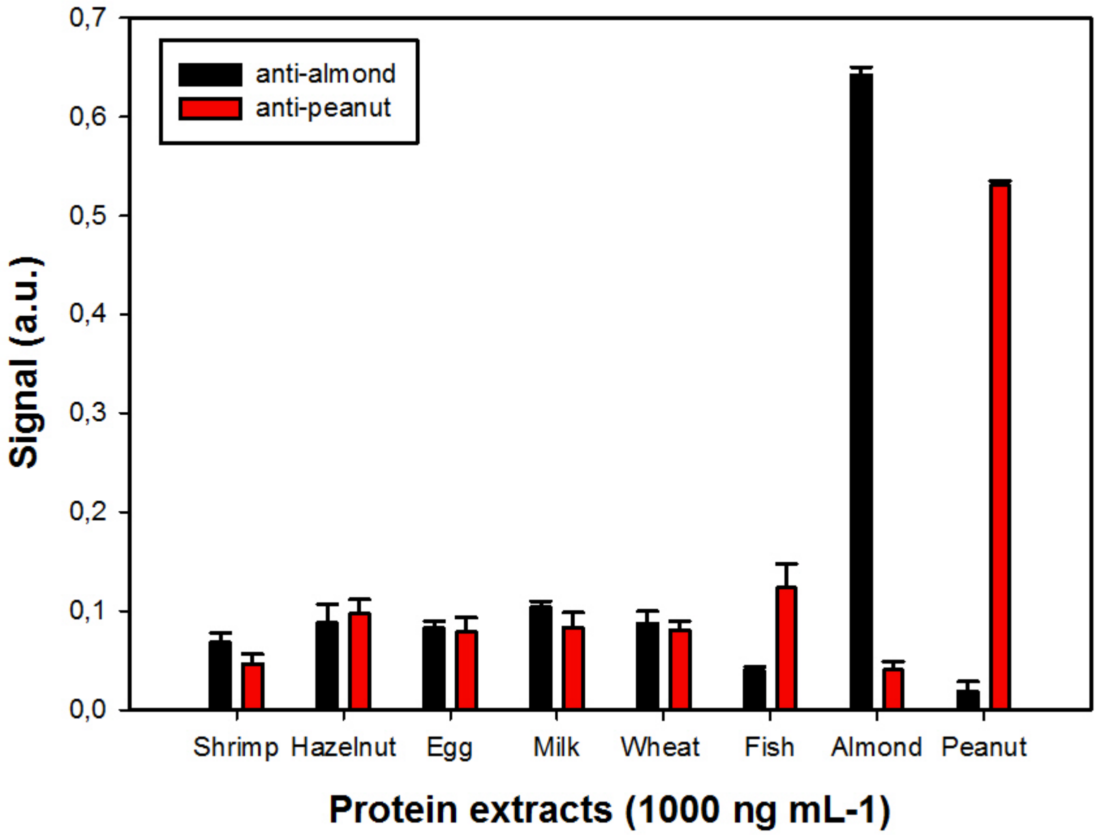

Figure S9. Normalized signal intensities achieved in the first test zone (row 1) of the LFμIA when analyzing 1000 ng mL^{-1} of shrimp, hazelnut, egg, milk, wheat, fish, almond, and peanut allergens.

Table S4. Cross-reactivity (%) of the almond and peanut assays when analyzing 1000 ng mL^{-1} of the prick test allergens.

Assay	Shrimp	Hazelnut	Egg	Milk	Wheat	Fish	Almond	Peanut
**Almond**	10.6	13.7	12.8	16.2	13.6	6.2	-	2.9
**Peanut**	8.8	18.3	14.8	15.6	15.2	23.3	7.6	-

Table S5. Limit of detection (LoD), Limit of quantification (LoQ), IC50, dynamic range (DR) and linear regression coefficient (r^2) of the internal calibration curve.

Assay	LoD (ng mL^{-1})	LoQ (ng mL^{-1})	IC_{50} (ng mL^{-1})	DR (ng mL^{-1})	r^2
**Almond**	153.6	183.7	556.7 ± 50.1	30-2563	0.99
**Peanut**	218.5	225.1	1089.6 ± 160.1	102-2581	0.99

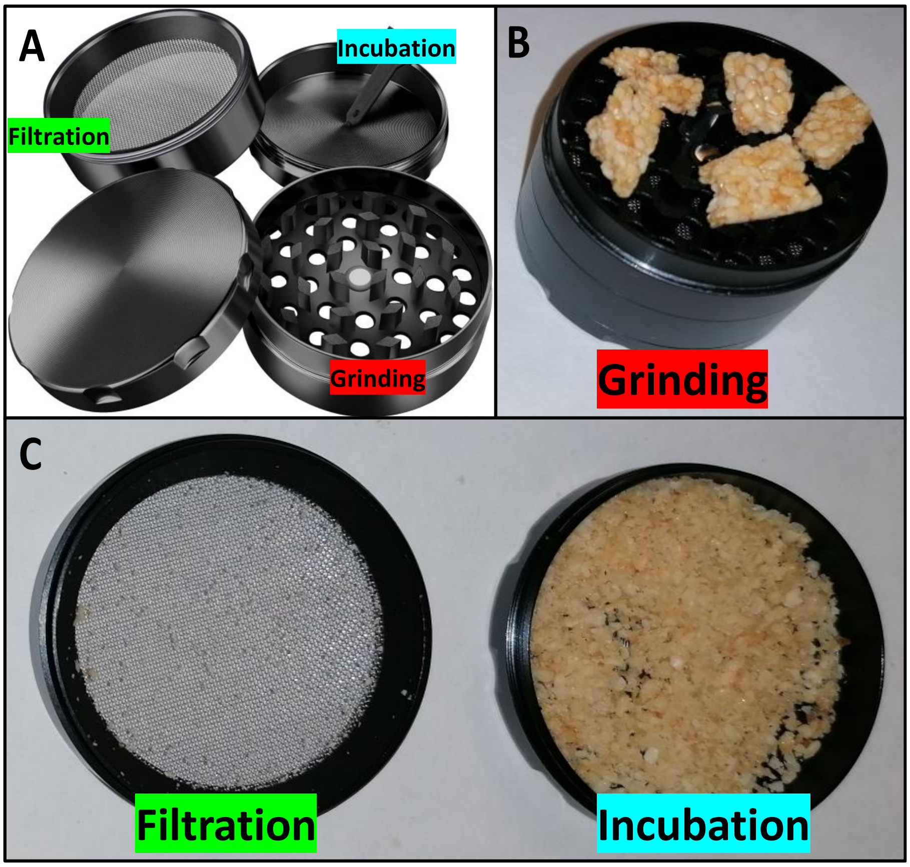

Figure S10. (A) Pictures of the proposed grinder-based extraction method. **(B)** The snack bar is grinded, **(C)** the shavings are filtered and incubated with the extraction buffer.

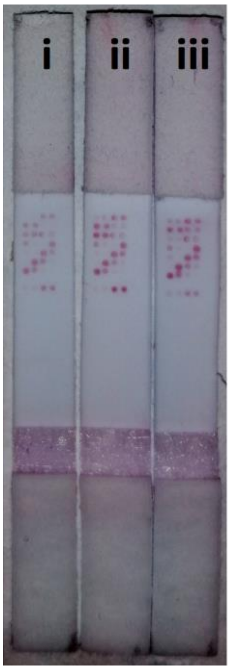

Figure S11. Pictures of the LFμIA strips after the detection of **(i)** 23000, **(ii)** 2300 and **(iii)** 230 ng mL^{-1} of the protein solution extracted from a snack bar.
